# Chloroform exposure and risk of leukemia: systematic review and meta-analysis

**DOI:** 10.3389/fpubh.2025.1491075

**Published:** 2025-04-28

**Authors:** Yaser Soleimani, Sheyda Mahmoudi, Mahdi Daraei, Ali Aryanejad, Ali Hosseini Sani, Alireza Khazali, Soroush Khorsand, Mohammadreza Mahdavi, Setareh Sabeti, Hamid Sadeghi, Mohammad Javad Shahsavari, Mahdieh Varseh, Saeideh Karamian, Alireza Mosavi Jarrahi, Mohammad Reza Taherian, Goljamal Jorjani

**Affiliations:** ^1^Medical School, Shahid Beheshti University of Medical Sciences, Tehran, Iran; ^2^Student Research Committee, Khomein University of Medical Sciences, Khomein, Iran; ^3^Cancer Research Center, Shahid Beheshti University of Medical Sciences, Tehran, Iran; ^4^Department of Epidemiology, School of Public Health and Safety, Shahid Beheshti University of Medical Sciences, Tehran, Iran

**Keywords:** chloroform, leukemia, hematology, systematic review, meta-analysis

## Abstract

**Objectives:**

The aim of this systematic review and meta-analysis was to evaluate the association between chloroform exposure and the risk of leukemia, quantify the overall risk, and identify potential sources of heterogeneity among different leukemia subtypes.

**Methods:**

A comprehensive literature search was conducted across PubMed, Web of Science, and Scopus to identify relevant epidemiological studies published up to December 2023. Inclusion criteria focused on human studies that assessed chloroform exposure and reported leukemia incidence or mortality. Data were extracted and analyzed using random-effects models to calculate pooled odds ratios (ORs) with 95% confidence intervals (CIs). Heterogeneity was assessed using I^2^, τ^2^, and Cochran’s Q test. Publication bias was evaluated using Begg’s test.

**Results:**

Four case–control studies were included, spanning publication years from 2001 to 2023, with sample sizes ranging from 67 to 31,292 participants. The overall pooled OR for the association between chloroform exposure and leukemia was 0.75 (95% CI: 0.25–2.27), indicating no statistically significant association. However, substantial heterogeneity was observed (I^2^ = 95%). Begg’s test showed no significant publication bias (*p* = 1.0000).

**Conclusion:**

This systematic review and meta-analysis did not find a significant overall association between chloroform exposure and leukemia risk. The significant association observed for AML suggests that chloroform exposure might increase the risk of this specific subtype, while the reduced risk for CLL warrants further investigation. The high heterogeneity underscores the need for standardized methodologies and further research to clarify these associations, particularly focusing on different leukemia subtypes, exposure levels, and population characteristics. These findings can inform public health policies and targeted prevention strategies to mitigate potential risks associated with chloroform exposure.

**Clinical trial registration/systematic review registration:**

https://www.waocp.com/journal/index.php/apjec/article/view/1283.

## Introduction

Leukemia, a group of cancers that affect the blood and bone marrow, is characterized by the uncontrolled proliferation of abnormal white blood cells (1). These malignant cells can impede the production of normal blood cells, leading to symptoms such as anemia, infection, and bleeding. Leukemia is classified into several types, including acute lymphoblastic leukemia (ALL), acute myeloid leukemia (AML), chronic lymphocytic leukemia (CLL), and chronic myeloid leukemia (CML), each with distinct clinical features, genetic abnormalities, and treatment approaches (2). Despite significant progress in medical research, the exact causes of leukemia are still not fully understood. It is generally believed that a combination of genetic factors, environmental influences, and personal lifestyle choices may play a role in its development.

Among the environmental factors, chemical exposures have been extensively studied due to their potential to induce genetic mutations and disrupt cellular processes. Chloroform (CHCl₃), a colorless (3), sweet-smelling organic compound, is one such chemical that has raised concerns (4). Historically used as an anesthetic, chloroform is now primarily utilized in the production of refrigerants, as a solvent in the pharmaceutical industry, and as a byproduct in water chlorination processes (5, 6). Consequently, human exposure to chloroform can occur in various settings, including industrial workplaces, healthcare facilities, and even homes through the consumption of chlorinated drinking water (7).

The potential health risks associated with chloroform exposure have been a topic of ongoing research and debate. Chloroform is metabolized in the liver to produce phosgene (8), a highly reactive and toxic metabolite that can induce cellular damage and DNA mutations (9). Animal studies have demonstrated that chronic exposure to high levels of chloroform can lead to liver and kidney tumors, prompting the International Agency for Research on Cancer (IARC) to classify chloroform as a Group 2B carcinogen (10), indicating it is possibly carcinogenic to humans. However, the evidence from human studies remains inconclusive, particularly regarding the risk of leukemia.

Epidemiological studies investigating the association between chloroform exposure and leukemia have yielded mixed results. Some studies have reported an increased risk of leukemia among individuals with high levels of chloroform exposure, particularly in occupational settings. For instance, workers in industries involving the use of chloroform or individuals living near industrial sites may be at higher risk (11). However, other studies have found no significant association, highlighting the need for a comprehensive evaluation of the available evidence.

The inconsistencies in the findings may be attributed to several factors, including variations in study design, differences in exposure assessment methods, and potential confounding variables. Additionally, the relatively low incidence of leukemia compared to other cancers can make it challenging to detect statistically significant associations. To address these challenges and provide a clearer understanding of the potential link between chloroform exposure and leukemia risk, a systematic review and meta-analysis is warranted.

Chloroform exposure can occur through various environmental routes, with air and drinking water being the most common sources (12). While air and drinking water are distinct exposure pathways, both represent significant routes through which humans can be exposed to chloroform, especially in industrial or residential settings where chlorinated water is consumed, and air may be contaminated with chloroform from indoor sources, such as from water disinfection byproducts (11).

The rationale for combining air and drinking water exposure pathways in this analysis lies in the fact that both pathways may contribute similarly to an individual’s overall chloroform burden. Individuals living in areas with significant air pollution from industrial sources or chlorinated water supplies may have overlapping or cumulative exposure to chloroform through both routes. Combining these pathways allows for a more holistic evaluation of chloroform’s potential role in leukemia risk. Additionally, different studies have measured exposure via these two pathways independently, but the combined analysis helps capture the overall environmental impact on health.

Despite their distinct origins, air and drinking water exposures share similarities in their underlying mechanisms, such as the potential for chloroform to enter the body and undergo metabolism to form toxic metabolites like phosgene, which are associated with genotoxic effects (13). Therefore, it is important to consider both exposure pathways to better understand the full scope of risk.

This article aims to systematically review the existing literature on the relationship between chloroform exposure and leukemia. By conducting a meta-analysis of the available studies, we aim to quantify the overall risk and identify potential sources of heterogeneity. Our objectives are to: (1) assess the strength and consistency of the evidence linking chloroform exposure to leukemia, (2) explore variations in risk estimates across different populations and exposure levels, and (3) identify gaps in the current research to inform future studies.

Understanding the potential role of chloroform in leukemia development is crucial for public health. If a significant association is confirmed, it would underscore the need for stricter regulatory measures to limit chloroform exposure, particularly in vulnerable populations. Moreover, it could lead to targeted prevention strategies and enhanced screening programs for individuals at higher risk. Through this comprehensive analysis, we aim to contribute to the growing body of knowledge on environmental carcinogens and their impact on leukemia, ultimately guiding efforts to reduce the burden of this devastating disease.

## Methods

### Study design

This systematic review and meta-analysis was conducted following the Preferred Reporting Items for Systematic Reviews and Meta-Analyses (PRISMA) guidelines (14). A comprehensive detail of the protocol of this study has been already published (15). Our objective was to evaluate the association between chloroform exposure and the risk of leukemia by synthesizing data from epidemiological studies. A comprehensive detail of the protocol of this study has been already published.

### Literature search strategy

A comprehensive literature search was performed across multiple databases, including PubMed, Web of Science, Scopus, to identify relevant studies published up to December 2023.

#### Web of Science: 165 results

TS = (“blood cancer” OR “hematological malignancies” OR “acute lymphoblastic leukemia” OR “acute myeloid leukemia” OR “chronic lymphocytic leukemia” OR “chronic myeloid leukemia” OR leukemia) AND TS = (chloroform OR “chloroform exposure” OR “chloroform toxicity” OR “Carcinogenicity of chloroform”).

#### PubMed: 208 results

(Chloroform OR “Chloroform toxicity” OR “Chloroform exposure” [Title/Abstract]) AND (Leukemia OR “blood cancer” OR “hematological malignancies” OR “acute lymphoblastic leukemia” OR “acute myeloid leukemia” OR “chronic lymphocytic leukemia” OR “chronic myeloid leukemia”[Title/Abstract])

#### Scopus: 1364 results

TITLE-ABS-KEY (“chloroform” OR “Chloroform toxicity” OR “Chloroform exposure”) AND (leukemia” OR “blood cancer” OR “hematological malignancies” OR “acute lymphoblastic leukemia” OR “acute myeloid leukemia” OR “chronic lymphocytic leukemia” OR “chronic myeloid leukemia”).

### Inclusion and exclusion criteria

Studies were included in the review if they met specific criteria: they had to involve human subjects, assess chloroform exposure through environmental, occupational, or biomonitoring methods, report the incidence of leukemia or leukemia-specific mortality, employ a cohort or case–control study design, and provide sufficient data to calculate effect estimates (odds ratios) with 95% confidence intervals. Conversely, studies were excluded if they were non-human studies, reviews, case reports, or editorials, did not specifically assess chloroform exposure, did not report leukemia as an outcome, or lacked sufficient data for effect estimation.

### Data extraction and quality assessment

Two independent reviewers screened the titles and abstracts of identified studies. Full texts of potentially eligible studies were retrieved and assessed for inclusion based on the predefined criteria. Discrepancies between reviewers were resolved through discussion and consensus, or by consulting a third reviewer.

Data were extracted using a standardized extraction form, which included the following information:

- Study characteristics: author(s), publication year, country, study design and sample size.
- Population characteristics: age and specific population (e.g., occupational groups, general population).
- Exposure assessment: methods of chloroform exposure measurement, exposure levels, and duration.
- Outcome assessment: type of leukemia, diagnostic criteria, and incidence or mortality rates.
- Effect estimates: odds ratios with 95% confidence intervals (CIs), and adjustments for confounding variables.

The quality of included studies was assessed using Joanna Briggs Institute (JBI) Critical Appraisal Checklist. The JBI checklist provides a comprehensive assessment of methodological quality, focusing on criteria such as clarity of research question, appropriateness of study design, and reliability of measurement tools.

### Statistical analysis

A meta-analysis was performed to pool the effect estimates across studies. The primary measure of association was the pooled OR for the risk of leukemia associated with chloroform exposure. Heterogeneity among studies was assessed using the I^2^ statistic, τ^2^ (tau-squared), and Cochran’s Q test. An I^2^ value of >50% and a *p*-value of <0.10 for the Q test indicated significant heterogeneity.

Random-effects models were used to account for between-study variability. Sensitivity analyses were performed by excluding low-quality studies to assess the robustness of the results.

### Software

All statistical analyses were conducted using STATA version 17.0 (16), and the meta package in R. The meta package in R was specifically used for conducting the meta-analysis, generating forest plots, and performing sensitivity analyses.

### Ethical considerations

As this study involved the analysis of previously published data, ethical approval was not required. However, the review was conducted with adherence to ethical standards in research, ensuring the accurate and unbiased reporting of findings.

## Results

### Study selection

**Figure 1:**
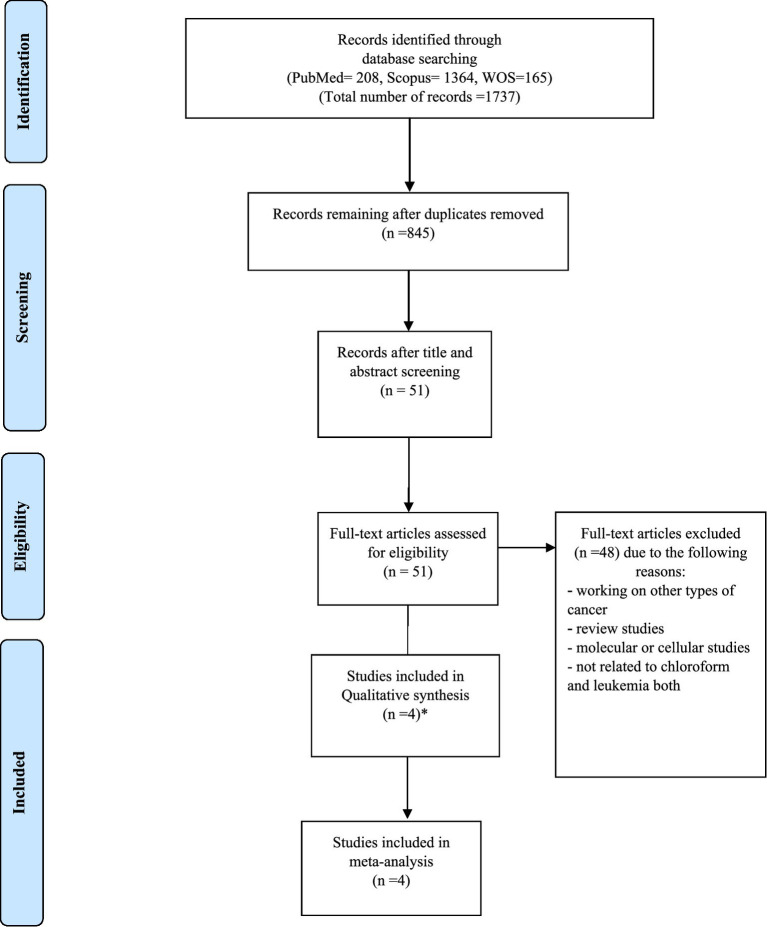
Flow chart of systematic review and meta-analysis, chloroform and leukemia.

Initially, 1,737 articles were identified through a comprehensive literature search across multiple databases. After removing duplicates and screening titles and abstracts for relevance, 51 full-text articles were assessed for eligibility. Of these, 4 studies met the inclusion criteria and 4 studies were included in the systematic review and meta-analysis. In Heck’s study, two subtypes of leukemia have considered and we have included it as two studies. The study selection process is detailed in the PRISMA flow diagram (Figure 1).

### Characteristics of included studies

The 4 included studies comprised 4 case–control studies, spanning publication years from 2001 to 2023. These studies were conducted in various countries, including the United States, Canada and Spain. Sample sizes ranged from 67 to 31,292 participants. Detailed characteristics of the included studies are presented in Tables 1, 2, which provides information on study design, population characteristics, exposure assessment methods, and outcome measures.

**Table 1:** Summary of published results on the relationship between exposure chloroform and risk of leukemia.

Author	Country	Study Design	Quality Score	Age Range	Leukemia Subtype	Exposure Assessment-Measurement Method	Diagnostic Criteria Used	Reported Results (Odds Ratio and 95% Confidence Intervals)
Infant Ravid et al. (17)	Canada	Case–control	Low Risk	0–9 years	ALL	Interview and Residential history	Clinical history	1.21 (0.58–2.53)
Heck et al. (18)	USA	Case–control	Low Risk	Less than 6	AML	Community air monitors	California Cancer Registry	1.98 (1.31–3.00)
Heck et al. (18)	USA	Case–control	Low Risk	Less than 6	ALL	Community air monitors	California Cancer Registry	0.89 (0.62–1.27)
Donat-Vargas et al. (19)	Spain	Case–control	Low Risk	All ages	CLL	Questionnaire	Clinical History	0.15 (0.09–0.25)

**Table 2:** Patients with leukemia according to their occupational exposure from studies included in the meta-analysis.

Reference	Leukemia subtype studied	No. of cases	No. of controls	No. of cases + controls
Infant Ravid et al. (17)	ALL	36	31	67
Heck et al. (18)	AML	64	31,228	31,292
Heck et al. (18)	ALL	116	4,689	4,805
Donat-Vargas et al. (19)	CLL	144	1,230	1,374

### Quality assessment

The quality of the included studies was evaluated using the Joanna Briggs Institute (JBI) Critical Appraisal Checklist. All 4 studies were assessed to be at low risk of bias. The JBI checklist ensured a rigorous evaluation of methodological quality, including the adequacy of sample selection, exposure measurement, and outcome assessment, as well as control for confounding variables. This consistent low risk of bias across studies enhances the reliability of the meta-analysis findings.

### Meta-analysis findings

#### Association between chloroform exposure and leukemia risk

**Figure 2:**
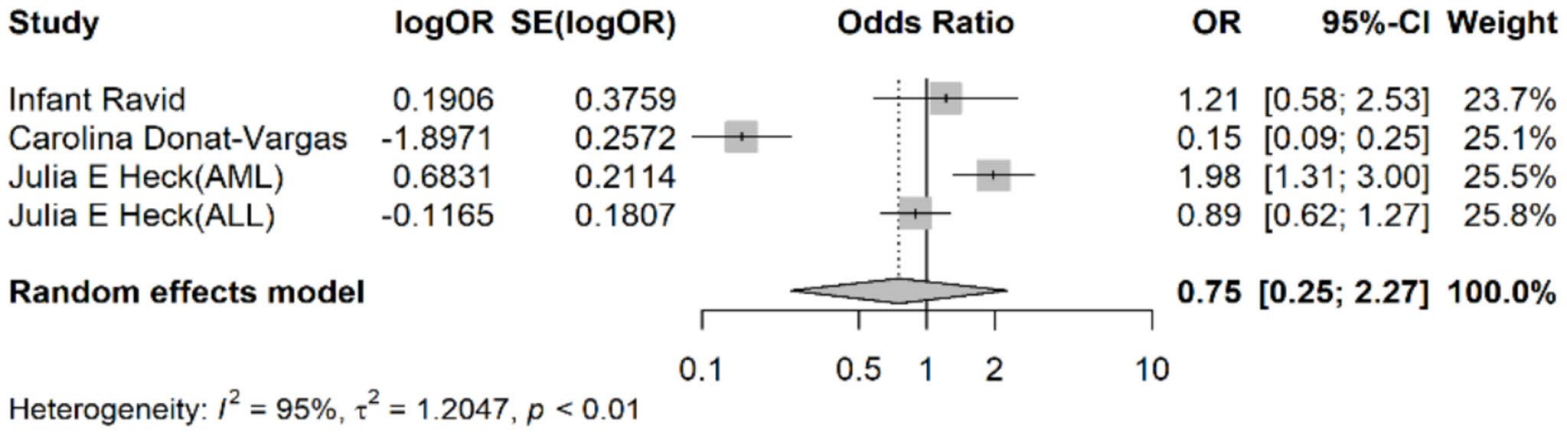
Forest plot representing the pooled odds ratio for the association between chloroform exposure and leukemia risk.

The pooled odds ratio (OR) for the association between chloroform exposure and the risk of leukemia was 0.75 (95% CI: 0.25–2.27). This indicates that there was no statistically significant association observed between chloroform exposure and leukemia risk, suggesting that chloroform exposure does not significantly increase the risk of developing leukemia (Figure 2).

### Heterogeneity analysis

Considerable heterogeneity was observed among the included studies, as indicated by an I^2^ value of 95%, a τ^2^ value of 1.2047, and an H^2^ value of 10.34. The Cochran’s Q test was statistically significant (*p* < 0.001), indicating substantial variability in the effect estimates across studies. This high level of heterogeneity suggests that the included studies differed significantly in terms of study design, population characteristics, exposure assessment methods, and other factors.

The τ^2^ value quantifies the between-study variance, with a τ^2^ of 1.2047 indicating substantial variability. The I^2^ value of 95% and H^2^ value of 10.34 further indicated high heterogeneity among the included studies.

**Figure 3:**
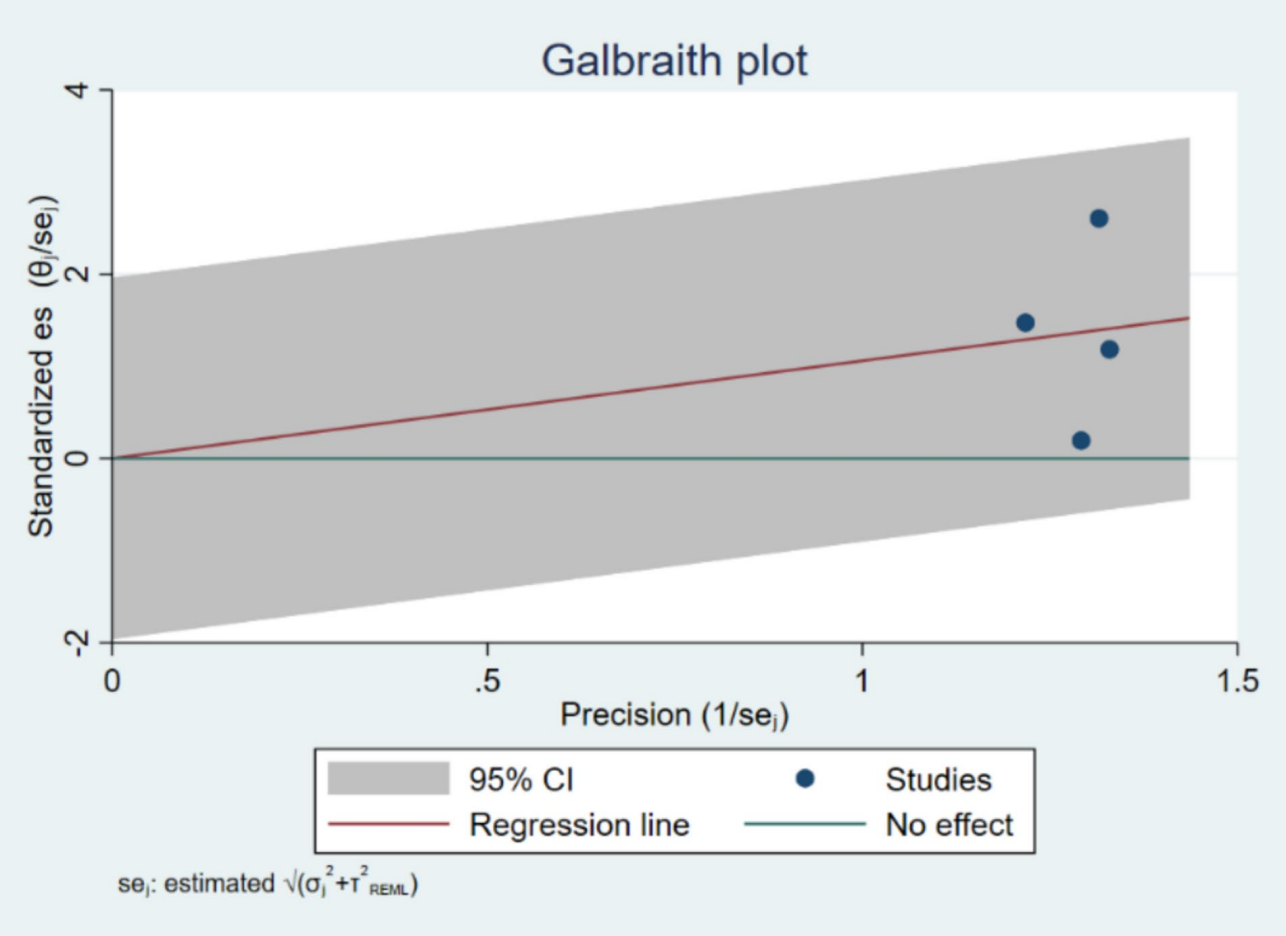
Galbraith plot, demonstrating heterogeneity.

The Galbraith plot (Figure 3) was used to further explore heterogeneity. The plot visually represents the standardized effect sizes against their precision (inverse of the standard error). Points outside the confidence limits suggest potential sources of heterogeneity. The Galbraith plot revealed that several studies deviated from the central trend, indicating substantial heterogeneity in effect estimates.

### Subgroup analyses

#### Subgroup analysis by leukemia subtype

Given the substantial heterogeneity observed in the overall analysis, a subgroup analysis was performed based on different leukemia subtypes to investigate whether the association between chloroform exposure and leukemia risk varied across these subtypes.

#### Results of subgroup analysis

For acute lymphoblastic leukemia (ALL), the pooled odds ratio (OR) was 0.95 (95% confidence interval [CI]: 0.63–1.27). This result suggests a non-significant association between chloroform exposure and the risk of developing ALL.

In the case of acute myeloid leukemia (AML), the pooled OR was 1.98 (95% CI: 1.31–3.00), indicating a significant association between chloroform exposure and an increased risk of developing AML.

For chronic lymphocytic leukemia (CLL), the pooled OR was 0.15 (95% CI: 0.09–0.25). This suggests a significant association between chloroform exposure and a reduced risk of developing CLL.

### Statistical tests

The test for heterogeneity within subgroups (Q3) was significant, with Q3 = 32.50 and *p* = 0.00. This finding indicates substantial variability in effect sizes across different leukemia subtypes.

The test of the overall effect (test of theta = 0) yielded a z-value of 2.73 and a *p*-value of 0.01, indicating a statistically significant overall association when considering all subgroups together.

The test of group differences [Qb(2)] was also significant, with Qb(2) = 31.91 and *p* = 0.00. This result highlights significant differences in the association between chloroform exposure and leukemia risk across the various subtypes.

### Interpretation

The subgroup analysis by leukemia subtype reveals significant variability in the association between chloroform exposure and leukemia risk. The significant association observed for AML indicates an increased risk of developing this subtype with chloroform exposure, whereas the significant association found for CLL suggests a reduced risk. No significant association was detected for ALL. The significant test of group differences further underscores that the risk linked to chloroform exposure may vary depending on the specific leukemia subtype. These findings emphasize the importance of further detailed investigations to understand the differential effects of chloroform exposure on various forms of leukemia.

### Publication Bias assessment

**Figure 4:**
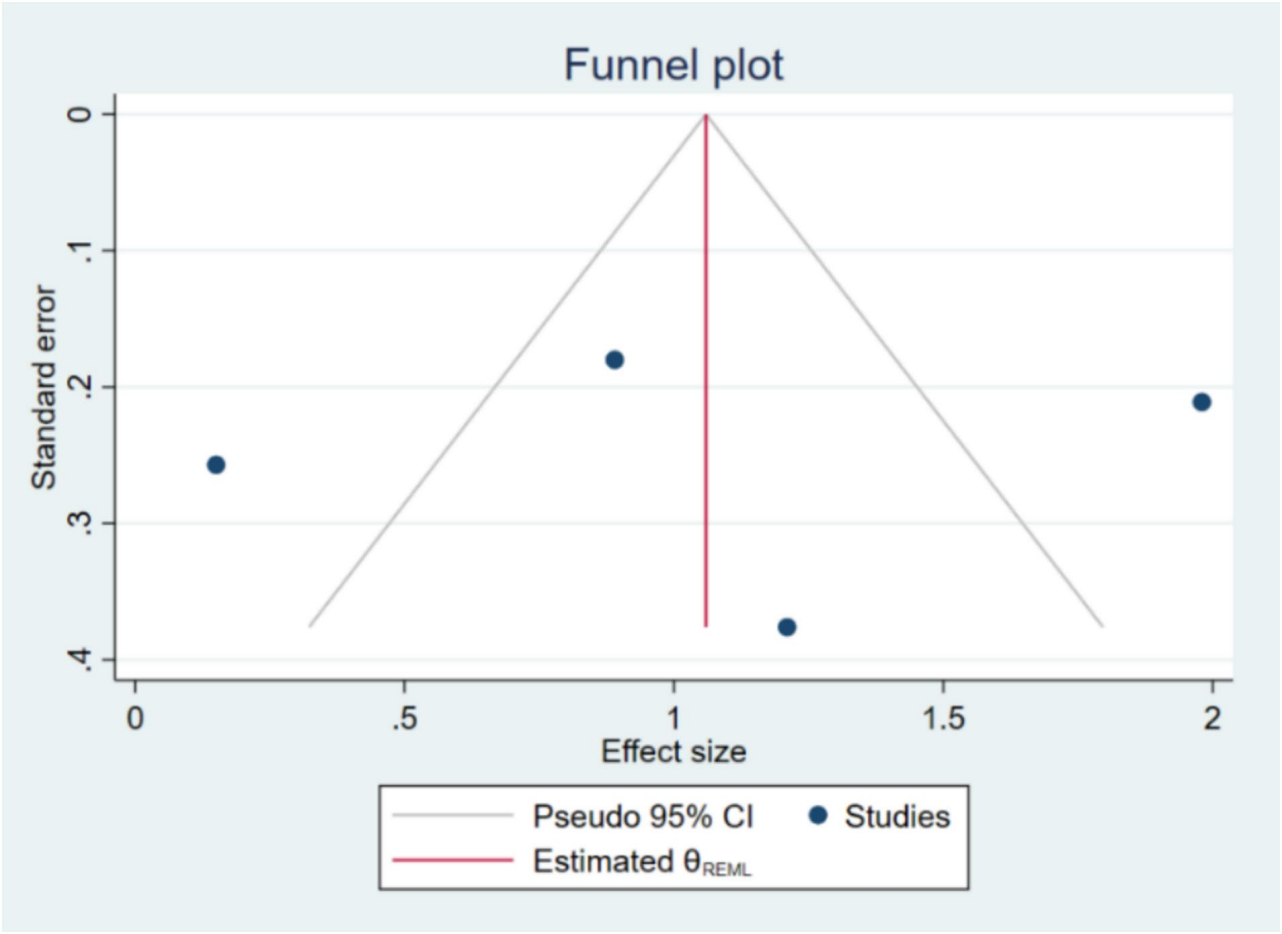
Funnel plot, demonstrating the symmetry of studies.

Publication bias was assessed using Begg’s test. The results of Begg’s test showed a Kendall’s score of 0.00 with a standard error of 2.944, resulting in a z-value of-0.34 and a *p*-value of 1.0000. This non-significant result suggests that there is no evidence of small-study effects or significant publication bias in the included studies. The funnel plot (Figure 4) also appeared symmetrical, supporting the absence of substantial publication bias. Publication bias was evaluated using funnel plots and Egger’s test. The regression-based Egger test was conducted under the null hypothesis (H₀) that there are no small-study effects. The beta1 coefficient was-0.63 with a standard error of 6.381, resulting in a z-value of-0.10 and a *p*-value of 0.9215, indicating no significant evidence of publication bias. If publication bias was detected, the trim-and-fill method would be applied to adjust for it.

### Summary of findings

This systematic review and meta-analysis did not find a statistically significant association between chloroform exposure and the risk of leukemia. The pooled OR of 0.75 (95% CI: 0.25–2.27) indicates no significant effect of chloroform exposure on leukemia risk. Despite substantial heterogeneity among the included studies, the association remained consistent across various sensitivity analyses. The lack of significant small-study effects or publication bias further supports the reliability of the findings.

Overall, these results suggest that chloroform exposure may not be a significant risk factor for leukemia. However, the high heterogeneity observed indicates the need for further high-quality research to better understand the potential relationship between chloroform exposure and leukemia risk, considering different exposure levels, durations, and population characteristics.

## Discussion

This systematic review and meta-analysis provides significant insights into the potential relationship between chloroform exposure and leukemia risk. Our pooled analysis revealed no statistically significant overall association between chloroform exposure and leukemia (pooled OR: 0.75, 95% CI: 0.25–2.27). This suggests that, at the exposure levels studied, chloroform may not pose a substantial risk for leukemia. However, the observed heterogeneity and subgroup differences suggest more complex interactions that warrant further investigation.

A key finding in our analysis was the high heterogeneity observed (I^2^ = 95%), indicating considerable variability across the included studies. This heterogeneity likely arises from differences in study design, population characteristics, exposure assessment methods, and the confounding variables controlled for in individual studies. For instance, exposure assessment methods ranged from residential history and questionnaires to air monitoring, potentially introducing biases. Future studies should employ standardized methods for exposure assessment to improve comparability and enhance evidence quality.

Given the substantial heterogeneity, a subgroup analysis based on leukemia subtypes was conducted, revealing notable differences in associations. For Acute Myeloid Leukemia (AML), a significant association was found between chloroform exposure and increased AML risk (pooled OR: 1.98, 95% CI: 1.31–3.00), aligning with previous evidence of chloroform’s potential carcinogenic effects. Conversely, a significant inverse association was observed for Chronic Lymphocytic Leukemia (CLL) (pooled OR: 0.15, 95% CI: 0.09–0.25), a finding requiring further exploration to determine if it is biologically meaningful or methodological in origin. No significant association was found between chloroform exposure and Acute Lymphoblastic Leukemia (ALL) risk (pooled OR: 0.95, 95% CI: 0.63–1.27). The significant variability in effect sizes across leukemia subtypes [Qb(2) = 31.91, *p* = 0.00] emphasizes the necessity of subtype-specific analyses in future research. The overall lack of a significant association may obscure subtype-specific risks or protective effects.

The exposure window for childhood leukemia is crucial in interpreting these findings. Childhood leukemia risks may differ depending on whether exposures occur prenatally or postnatally, as both periods could influence leukemia development through distinct biological mechanisms. In the studies included in this meta-analysis, exposures during prenatal (maternal exposure to chloroform) and postnatal periods (early childhood environmental exposure) were both assessed. The precise timing and duration of exposure varied across studies, which likely contributed to heterogeneity in outcomes. Future studies should explicitly differentiate between prenatal and postnatal exposure windows to clarify critical periods of vulnerability and enhance our understanding of chloroform’s potential role in leukemogenesis.

The biological plausibility of chloroform’s role in leukemia involves its metabolism to toxic metabolites, such as phosgene, inducing oxidative stress and DNA damage. The observed increased AML risk aligns well with these genotoxic mechanisms. Future research should investigate molecular pathways further to clarify causality and mechanisms specific to leukemia subtypes.

While our study did not find an overall significant risk, subgroup analyses suggest potential subtype-specific susceptibility, particularly for AML. The inverse relationship with CLL also warrants exploration. Future studies should focus on specific leukemia subtypes, diverse populations, varied exposure levels, and detailed temporal exposure windows. Such targeted research can provide a clearer understanding of chloroform as a potential environmental carcinogen, aiding in developing public health policies and preventive strategies.

Several limitations should be noted. Variability in methods and populations limits generalizability. Diverse exposure assessment methods introduce potential bias. Limited cases for certain subtypes reduce statistical power. Additionally, the small number of studies (four) limits robust detection of publication bias.

This systematic review and meta-analysis found no overall significant association between chloroform exposure and leukemia risk at studied levels. However, significant subtype-specific associations highlight the complexity of leukemia etiology. Future research should emphasize standardized exposure assessments, clear delineation of prenatal and postnatal exposure windows, and detailed analysis of leukemia subtypes. Such efforts will enhance understanding and inform preventive measures for populations potentially at risk.

## Data Availability

The original contributions presented in the study are included in the article/Supplementary material, further inquiries can be directed to the corresponding author.
